# EFFICACY OF PROBIOTICS IN PREVENTING CHEMOTHERAPY-INDUCED DIARRHEA IN GASTROINTESTINAL CANCER PATIENTS

**DOI:** 10.1590/S0004-2803.24612025-020

**Published:** 2025-12-01

**Authors:** Gabriel Caio DE SOUZA, Humberto Bezerra de Araujo, Cleyton Zanardo DE OLIVEIRA, Ana Paula Ribeiro PAIOTTI, Nora Manoukian FORONES

**Affiliations:** 1 Universidade Federal de São Paulo, Escola Paulista de Medicina, Departamento de Medicina, Programa de Pós-Graduação em Medicina Translacional, São Paulo, SP, Brasil.; 2 Universidade Federal de São Paulo, Escola Paulista de Medicina, Departamento de Medicina, Disciplina de Gastroenterologia Pediátrica, São Paulo, SP, Brasil.; 3 Ensino e Pesquisa, BP - A Beneficência Portuguesa de São Paulo, São Paulo, SP, Brasil.; 4 Universidade Federal de São Paulo, Escola Paulista de Medicina, Departamento de Medicina, Setor de Oncologia, Disciplina de Gastroenterologia, São Paulo, SP, Brasil.

**Keywords:** Diarrhea, antineoplastic agents, probiotics, Diarreia, antineoplásicos, probióticos

## Abstract

**Background:**

Chemotherapy-induced diarrhea is a common and distressing side effect experienced by patients undergoing cancer treatment, particularly those with gastrointestinal cancer. It can lead to significant health complications, including dehydration, electrolyte imbalances, and treatment interruptions. Recent studies have shown that the gut microbiome plays an important role in the development and severity of chemotherapy-induced diarrhea. Modulating the gut microbiome with probiotics has emerged as a potential strategy for preventing and managing chemotherapy-induced diarrhea.

**Objective:**

In this study we aimed to evaluate the efficacy of one probiotic containing a mixture of several strains of *Lactobacillus* and *Bifidobacterium* species in prevention of chemotherapy induced diarrhea among patients with gastrointestinal cancer.

**Methods:**

Between April 2022 and June 2024, a total of 28 patients diagnosed with gastrointestinal cancer who were intended to receive chemotherapy based on fluoropyrimidine, oxaliplatin, and/or irinotecan were randomized in a ratio 1:1 to receive either a placebo or 20 billion colony-forming units (CFU) of a mixture containing five viable strains including 335 mg of *Lactobacillus acidophilus* NCFM®, *Lactobacillus paracasei* Lpc-37TM, *Bifidobacterium lactis* Bi-04TM, *Bifidobacterium lactis* Bi-07TM, and *Bifidobacterium bifidum* Bb-02TM. Patients were instructed to take the product orally once daily for 90 days and to record their bowel habits in a diary using the Bristol stool scale.

**Results:**

The use of probiotics, compared to placebo, did not result in reduction of grade 2/3 diarrhea episodes (placebo arm 55.56% vs probiotic arm 44.44%; *P*=1). Likewise, no statistically significant difference was observed in the overall incidence of diarrhea between the two groups (71.43% vs 64.29%; *P*=1). The median number of diarrhea episodes during the 90-day follow-up tended to be lower in the probiotic group (eight episodes) compared to the placebo group (9 episodes) (*P*=0.639) Subgroup analyses failed to identify any specific patient characteristics that associated any benefit from the probiotic use, regardless of diarrhea grade. Also, no infections related to the probiotic strains administered in this study were detected.

**Conclusion:**

Probiotic in comparison to a placebo did not result in a statistically significant effect, suggesting a lack of benefit of administered probiotic for prevention of chemotherapy induced diarrhea among patients with gastrointestinal cancer.

## HIGHLIGHTS

• Chemotherapy-induced diarrhea (CID) is a common adverse event during the treatment for gastrointestinal cancers.

• Management of CID usually involves the use of antidiarrheal agents such as loperamide; however, few measures are effective in preventing its occurrence.

• Emerging evidence has demonstrated the role of gut microbiota in the pathophysiology of CID, suggesting that probiotics may be effective as a preventive approach.

## RESUMO

**Contexto:**

A diarreia induzida por quimioterapia é um efeito colateral comum e debilitante experimentado por pacientes em tratamento contra o câncer, particularmente aqueles com câncer gastrointestinal. Pode levar a complicações de saúde significativas, incluindo desidratação, desequilíbrios eletrolíticos e interrupções no tratamento. Estudos recentes mostraram que o microbioma intestinal desempenha um papel importante no desenvolvimento e na gravidade da diarreia induzida por quimioterapia. A modulação do microbioma intestinal através do uso de probióticos emergiu como uma estratégia potencial para a prevenção e manejo da diarreia induzida por quimioterapia.

**Objetivo:**

Neste estudo, nosso objetivo foi avaliar a eficácia de um probiótico contendo uma mistura de cepas das espécies de *Lactobacillus* e *Bifidobacterium* na prevenção da diarreia induzida por quimioterapia em pacientes com câncer gastrointestinal.

**Métodos:**

Entre abril de 2022 e junho de 2024, um total de 28 pacientes diagnosticados com câncer gastrointestinal que deveriam receber quimioterapia baseada em fluoropirimidina, oxaliplatina e/ou irinotecano foram randomizados na proporção de 1:1 para receber placebo ou 20 bilhões de unidades formadoras de colônia (CFU) de uma mistura contendo cinco cepas viáveis, incluindo 335 mg de *Lactobacillus acidophilus* NCFM®, *Lactobacillus paracasei* Lpc-37™, *Bifidobacterium lactis* Bi-04™, *Bifidobacterium lactis* Bi-07™ e Bifidobacterium bifidum Bb-02™. Os pacientes foram instruídos a tomar o produto oralmente uma vez ao dia por 90 dias e a registrar seus hábitos intestinais em um diário usando a escala de fezes de Bristol.

**Resultados:**

O uso de probióticos comparado ao placebo não resultou na redução de episódios de diarreia grau 2/3 (grupo placebo 55.56% vs grupo probiótico 44.44%; *P*=1). De mesma forma, nenhuma diferença estatisticamente significativa foi observada na incidência global de diarreia entre os dois grupos (71.43% vs 64.29%; *P*=1). A mediana de números de episódios de diarreia durante os 90 dias de acompanhamento apresentou uma tendência a ser menor no grupo probiótico (oito episódios) quando comparado ao grupo placebo (*P*=0.639). A análise de subgrupo falhou em identificar alguma característica específica que estivesse associada a qualquer benefício do uso do probiótico. Nenhuma infecção associada às cepas probióticas administradas no estudo foi detectada.

**Conclusão:**

O probiótico em comparação ao placebo não resultou em um efeito estatisticamente significativo no controle da diarreia, sugerindo a falta de benefício do probiótico administrado para a prevenção da diarreia induzida por quimioterapia em pacientes com câncer gastrointestinal.

## INTRODUCTION

Gastrointestinal (GI) cancers represent 25% of all cancer cases and 33% of cancer-related deaths in the whole world. The Incidence and mortality rates can vary significantly worldwide. While gastric cancer rates have decreased in some areas, colorectal cancer cases have risen in regions where it was previously low. Moreover, high income regions are witnessing an increase, in liver and pancreatic cancer cases^1^.

Chemotherapy-induced diarrhea (CID) is an important and frequent adverse effect of cancer treatment, especially in patients diagnosed with GI cancer. The prevalence of CID depends on the chemotherapeutic drugs and patient factors. For instance, regimens including 5-fluorouracil (5-FU), capecitabine, and irinotecan are associated with a higher risk for developing CID, with reported incidences ranging from 30% to 80% of patients^2^ ^,^ ^3^. In some cases, CID can lead to grade 3 or 4 toxicity, characterized by more than seven bowel movements per day or life-threatening complications, which occur in more than 30% of patients receiving chemotherapy^3^.

Patients with GI tract cancers are particularly vulnerable to CID due to the impact of tumor-related disruption of gut function and the cytotoxic effects of chemotherapy on rapidly dividing epithelial cells in the intestinal mucosa^4^. This cytotoxicity results in a breakdown of the gut barrier, increased permeability, and an inflammatory cascade, all of which exacerbate diarrhea^5^. Additionally, chemotherapy-induced dysbiosis, or an imbalance in the gut microbiota, further aggravates mucosal injury and inflammation^6^.

The clinical implications of CID are very important. Diarrhea not only diminishes patients’ quality of life but also interrupts treatment cycles, may need dose reductions, and increases the risk of hospitalization and healthcare costs^5^. These interruptions can compromise the efficacy of cancer treatment and impact on the overall survival^5^. Current management strategies, such as loperamide and octreotide, focus primarily on symptomatic relief but fail to address the underlying pathophysiological changes^7^. Moreover, these treatments can have their own side effects, including constipation, nausea, and abdominal cramping, further complicating patient care.

Probiotics, defined as live microorganisms that confer health benefits when administered in adequate amounts, have emerged as a promising adjunctive therapy for managing CID^8^. Many genera and phylum of bacteria are shown to possess probiotic qualities; however, the *Lactobacillus* and *Bifidobacterium* species are most employed as probiotics^8^. The mechanism of action of probiotics in mitigate CID may take multiple pathways, including the restoration of gut microbiota balance, enhancement of intestinal barrier integrity, and modulation of immune responses^9^ ^,^ ^10^. Preclinical and clinical studies have shown that certain strains of *Lactobacillus* and *Bifidobacterium* can reduce the severity and duration of diarrhea, decrease markers of inflammation such as TNF-α and IL-6, and improve overall gut health in patients undergoing chemotherapy^10^.

Even with these promising findings, the evidence remains uncertain due to heterogeneity in study designs, probiotic strains, dose, and patient populations. This highlights the need for more well-designed randomized controlled trials to establish the efficacy and safety of probiotics in preventing CID in specific cancer subgroups, particularly in patients with GI cancer. In the study we aimed to investigate the role of one probiotic (mixed of several strains of *Lactobacillus* and *Bifidobacterium* species) as a preventive strategy for CID.

## METHODS

This prospective study was conducted at the Clinical Oncology Division of the Universidade Federal de São Paulo (Brazil). Between April 2022 and June 2024, 28 patients with confirmed diagnosis of gastrointestinal tract tumors and intended to begin chemotherapy treatment containing at least one fluoropyrimidine and/or irinotecan were recruited and randomized to receive a probiotic containing 20 billion colony-forming units (CFU) of *Lactobacillus* and *Bifidobacterium* genera. Participants had their bowel habits monitored over a period of 90 days to evaluate the number of diarrhea episodes.

The study was conducted in accordance with the World Medical Association (WMA) and the Declaration of Helsinki. Ethical approval for this study was obtained from the Ethics and Clinical Research Committee Coordenadoria de Ensino e Pesquisa do Hospital São Paulo-HU/UNIFESP registered under the number CAAE: 51125421.5.0000.5505 and Universal Trial Number (UTN): U1111-1296-2263. Patients provided and signed written informed consent prior to their inclusion.

### Study population

Eligible patients were aged 18 years or older, with histologically confirmed primary gastrointestinal tract tumors (stomach, pancreas, colon, and rectum), and planned to initiate chemotherapy with a regimen containing fluoropyrimidine and/or irinotecan, regardless the line of treatment. Performance status was assessed using the Eastern Cooperative Oncology Group (ECOG) scale and limited to 0-2.

Exclusion criteria included patients who had received chemotherapy within three months before the randomization; those with severe systemic diseases, such as heart disease, coagulopathies, or renal and hepatic dysfunction; a history of severe diarrhea prior treatment; use of probiotics or prebiotics within two months before recruitment; pregnant or breastfeeding women; patients unable to provide consent; patients using a colostomy and those who declined to sign the Informed Consent Form (ICF). 

### Trial design

This is a randomized, double-blind study that aimed to evaluate the efficacy of the probiotic 20Bi® in the reduction of total number of diarrhea events compared to placebo. The randomization was conducted by the Núcleo de Apoio e Incentivo ao Pesquisador da BP - A Beneficência Portuguesa de São Paulo, which generated a random sequence (using R software) considering two treatments (Probiotic vs Placebo) and blocks of 2, 4, and 6 (3 of each). This sequence was subsequently implemented in REDcap. Patients were randomized in a 1:1 ratio. The containers with probiotic/placebo had the same appearance and were randomly numbered. As a double-blind study, only the pharmacy staff that produced the placebos and a member of the Núcleo de Apoio e Incentivo ao Pesquisador had access to the randomization guide. Other members of the project were unaware of which treatment the patient received.

### Intervention

The probiotic used in this study was 20bi® formula (produced by Momenta Farmacêutica, Brazil) that included a mix of five viable strains in a 335 mg capsule: *Lactobacillus acidophilus* NCFM®, *Lactobacillus paracasei* Lpc-37™, *Bifidobacterium lactis* Bi-04™, *Bifidobacterium lactis* Bi-07™, and *Bifidobacterium bifidum* Bb-02™. The placebo was produced to be indistinguishable from the probiotic in terms of color, appearance, taste, size, and shape, containing only pharmaceutical talc (magnesium silicate). Patients were instructed to take one capsule of probiotic/placebo once daily, preferably after a meal, for a period of 90 days. The capsule should be swallowed whole and not opened. The intervention with probiotic/placebo administration started one week prior to the beginning of the first chemotherapy cycle.

Participants receiving irinotecan were managed according to standard protocols for the prevention of early-onset diarrhea, which included a premedication dose of atropine sulfate (0.25 mg to 1 mg) administered immediately before irinotecan infusion. For late-onset diarrhea associated with chemotherapy, all participants received instructions to use loperamide, starting with an initial dose of 4 mg followed by 2 mg every 4 hours or after each subsequent diarrhea episode.

### Treatment evaluation

To evaluate the primary outcome, diarrhea, patients were instructed to maintain a diary and register the daily number of evacuations and stool consistency. For this purpose, an adapted and validated Portuguese version of bristol stool form scale was used^11^, which describes seven stool types. Diarrhea was defined as the occurrence of types 6 and 7 according to the scale:

Type 1: separate hard lumps, like nuts (hard to pass).

Type 2: sausage-shaped but lumpy.

Type 3: like a sausage but with cracks on its surface.

Type 4: like an Italian sausage or snake, smooth and soft.

Type 5: soft blobs with clear-cut edges (passed easily).

Type 6: fluffy pieces with ragged edges, a mushy stool.

Type 7: watery, no solid pieces. Entirely liquid.

To graduate diarrhea toxicity, the NCI common terminology criteria for adverse events version 4.1 (CTCAE) was applied. 

Additional clinical and demographic data were collected to correlate with episodes of diarrhea. These included age, gender, race, primary tumor location (target organ and upper or lower gastrointestinal tract), body mass index, presence of metastases at randomization, cancer staging, and treatment regimen.

### Statistical analysis

The statistical methodology employed in the evaluation involved data description using measures such as mean, standard deviation, minimum and maximum values, and quartiles for numerical variables, as well as absolute and relative frequencies for categorical variables. When appropriate, 95% confidence intervals were calculated.

The homogeneity between intervention groups (placebo and probiotic) concerning patients’ demographic and clinical characteristics was assessed using Fisher’s exact test for categorical variables and the Mann-Whitney test for numerical variables.

Initially, the relationship between patients’ demographic and clinical characteristics and the grade of diarrhea toxicity within each intervention group was examined using Fisher’s exact test for categorical variables and the Mann-Whitney test for numerical variables. Subsequently, to investigate the combined relationship, a logistic regression model was applied, along with the calculation of odds ratios and their respective confidence intervals.

Throughout all phases of the study, a significance level of 0.05 was adopted. The analyses were conducted using R software.

## RESULTS

**FIGURE 1:**
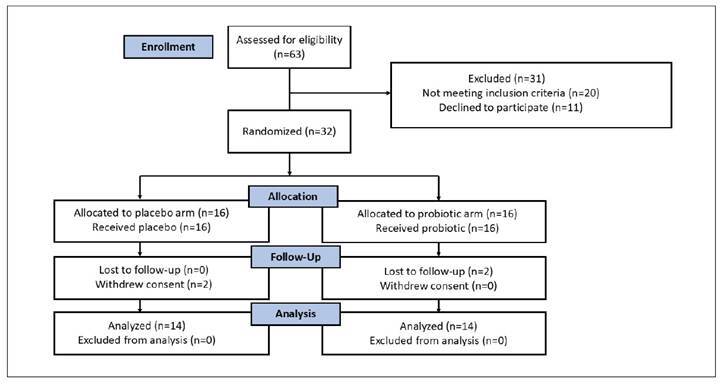
CONSORT flow diagram.

Between April 2022 and June 2024, 28 patients were randomized in a 1:1 ratio. A total of 63 potential participants were assessed for eligibility. Among these, 20 individuals did not meet the inclusion criteria, and 11 eligible patients declined to participate due to the complexity of registering their bowl habits in the diary, lack of interest in the study or language barriers (one patient). Eventually, 32 participants were randomized, of whom 28 completed the study, from which 14 were allocated to the probiotic and 14 to the placebo arm, respectively. The CONSORT diagram summarizing participant flow is presented in Figure 1.

Patient’s baseline characteristics, including age, sex, tumor location, and chemotherapy regimens were described on are presented in Table 1. The distribution of baseline characteristics was generally homogeneous between arms regarding age, gender, and primary tumor location. However, the placebo group included a single patient with pancreatic cancer, a tumor not represented in the probiotic group. Tumor location did not differ significantly when categorized as upper gastrointestinal tract versus lower gastrointestinal tract. In the probiotic group, a higher proportion of patients presented with Stage III disease (42.86%) compared to the placebo group (21.43%). However, both groups had the same number of patients with metastatic disease (n=6; 42.86%). In both arms, most patients received a combination of capecitabine and oxaliplatin (CAPOX). In contrast, irinotecan-based regimens were less commonly prescribed, though they were slightly more frequent in the probiotic arm (14.28%) compared to the placebo arm (7.14%). This distribution reflects the predominance of fluoropyrimidine and oxaliplatin combinations as the standard treatment choice within the study cohort. Patients with a body mass index (BMI) over 25 were slightly more common in the placebo group.

**TABLE 1:** Patient’s baseline characteristics.

Variables	Placebo		Probiotic		***P*-value**
	N	%	N	%	
**All patients**	14	50.00	14	50.00	
**Age, median**					
(range), years	61	(52-84)	57	(32-72)	0.08
**Gender**					
Female	7	50.00	6	42.86	1
Male	7	50.00	8	57.14	
**Tumor site**					
Colon	4	28.57	2	14.29	0.836
Duodenum	1	7.14	1	7.14	
Stomach	3	21.43	5	35.71	
Pancreas	1	7.14	0	0.00	
Rectum	5	35.71	6	42.86	
**GI**					
Lower	9	64.29	8	57.14	1
Upper	5	35.71	6	42.86	
**Staging**					
I	1	7.14	0	0.00	0.482
II	4	28.57	2	14.29	
III	3	21.43	6	42.86	
IV	6	42.86	6	42.86	
**BMI**					
< 25	8	57.14	10	71.43	0.695
≥ 25	6	42.86	4	28.57	
**Treatment**					
Capecitabine	3	21.43	1	7.14	0.733
CAPOX	8	57.14	7	50.00	
FLOT	0	0.00	1	7.14	
FLOX	2	14.29	3	21.43	
FOLFIRI	0	0.00	1	7.14	
FOLFIRINOX	1	7.14	0	0.00	
IFL	0	0.00	1	7.14	

BMI: body mass index. GI: gastrointestinal tract. 5FU+OX: fluoropirimidine+oxaliplatin. CAPOX: capecitabine + oxaliplatin. FLOX: fluoruracil + calcium folinate + oxaliplatin. FLOT: fluoruracil + calcium folinate + oxaliplatin + docetaxel. FOLFIRINOX: fluoruracil + calcium Folinate + oxaliplatin + irinotecan. FOLFIRI / IFL: fluoruracil + calcium folinate + irinotecan.

The absence of diarrhea was observed in 28.6% of patients in the placebo group and 35.7% in the probiotic group. Rates of Grade 1 and 2 diarrhea were identical across groups (35.7% and 21.4%, respectively). Severe diarrhea (Grade 3) was reported in two patients in the placebo group and one in the probiotic group (14.3% vs 7.1%, respectively). There were no cases of Grade 4 diarrhea in either group (Table 2).

**TABLE 2:** Diarrhea rate by grade.

Diarrhea	Placebo		Probiotic		***P*-value**
	N	%	N	%	
Absence	4	28.57	5	35.71	1
Grade 1	5	35.71	5	35.71	
Grade 2	3	21.43	3	21.43	
Grade 3	2	14.29	1	7.14	

The overall incidence of diarrhea was lower in probiotic group; however this difference was not statistically significant (placebo arm 71.43% vs probiotic arm 64.29%, *P*=1). The median number of diarrhea episodes during the 90-day follow-up tended to be lower in the probiotic group in the probiotic group (8 episodes) compared to the placebo group (*P*= 0.639) (Table 3).

**TABLE 3:** Incidence of diarrhea - number of episodes during follow-up.

Arm	N	Mean	Median	Max. Number of episodes	***P*-value**
Placebo	14	22	9	100	0.639
Probiotic	14	22	8	209	

An analysis grouping diarrhea grades 2 and 3 also did not demonstrate any significant difference between the intervention and the placebo arms, even though the probiotic arm presented lower rates of diarrhea occurrence (44.44% vs 55.56%) (Table 4)

**TABLE 4:** Diarrhea rate by grade - grouped.

Diarrhea	Placebo		Probiotic		***P*-value**
	N	%	N	%	
Absence	4	44.44	5	55.56	1
Grade 1	5	50	5	50	
Grade 2/3	5	55.56	4	44.44	

A multivariate analysis was performed to assess potential associations between diarrhea risk and subgroup characteristics, including sex, chemotherapy regimen, metastatic status, and body mass index. None of these factors were statistically significant predictors of diarrhea incidence (Table 5). No infection caused by probiotic strains was observed in this study.

**TABLE 5:** Multivariate analysis of the subgoups.

	***P*-value**	OR (95%CI)
Probiotic	0.69	1.39 (0.28 - 6.84)
Age (years)	0.15	0.93 (0.85 - 1.03)
Gender	0.51	1.72 (0.35 - 8.51)
Metastatic	0.49	1.80 (0.34 - 9.40)
GI	0.21	3.15 (0.51 - 19.27)
BMI	0.86	1.17 (0.22 - 6.20)
5FU + OX	0.30	2.67 (0.42 - 17.05)

OR: Odds Ratio.

## DISCUSSION

This clinical trial failed to achieve its primary endpoint of reducing the overall incidence of chemotherapy-induced diarrhea through the administration of probiotics tested. Despite preclinical and clinical evidence suggesting the potential benefits of probiotics in mitigating CID^12^ ^,^ ^13^, our randomized controlled trial did not find any statistically significant difference in the incidence of CID, in any grade, between the probiotic and placebo groups. Moreover, the study was not able to identify any subgroups of patients who might benefit from probiotic administration.

There is not a consensus that probiotics can prevent CID in patients on chemotherapy. Some previous studies suggested that certain probiotic strains, particularly those from the genera *Bifidobacterium* and *Lactobacillus*, could play a protective role in preventing CID^13^ ^-^ ^16^.

Some meta-analyses and randomized controlled trials have reported different degrees of efficacy for probiotics in the prevention of CID. A meta-analysis by Lu et al. (2019) included 13 randomized controlled trials (RCTs), 3 of them placebo controlled, and concluded that probiotic supplementation reduced the overall incidence, severity of diarrhea and occurrence of grade 3 or higher, but showed no effect on the occurrence rate of grade 1-2 diarrhea, corroborating our findings for occurrence of lower grades of diarrhea^10^.

A randomized study conducted by Mego et al. (2015) evaluating the efficacy of a probiotic formula containing Bifidobacterium bifidum and Lactobacillus acidophilus^13^, strains also present in our formulation, reported a reduction in the incidence of severe irinotecan-induced diarrhea and a trend toward reduced overall diarrhea in patients with colorectal cancer. However, in a subsequent phase III trial with a larger cohort, the same group (Mego et al., 2023) tested a different probiotic combination (*Bifidobacterium animalis subsp. lactis* BB-12® and *Lactobacillus rhamnosus* LGG®) and found no statistically significant benefit in reducing the overall incidence of irinotecan-induced diarrhea^17^. This supports the idea that probiotic efficacy can be inconsistent and influenced by multiple factors, including gut microbiome composition, chemotherapy-induced dysbiosis, and individual patient responses.

The composition and dosing of the probiotic used in this study also demands further exploration. The formulation used in the study included a high concentration of strains, totaling 20 billion colony-forming units (CFU) per dose (2×10¹º CFU). While this appears robust, it is possible that a richer array of strains administered in smaller concentrations might yield different outcomes. Prior studies have suggested that certain strains, such as *Lactobacillus rhamnosus* GG (LGG®) and *Bifidobacterium* BB-12®, may be more effective in modulating gut microbiota and improving intestinal barrier function^18^ ^,^ ^19^.

Certain chemotherapy agents, such as irinotecan, have been shown to induce diarrhea through mechanisms involving bacterial beta-D-glucuronidase activity, which may be modulated by probiotics^13^. However, fluoropyrimidine and oxaliplatin-based regimens, predominant in our study, may have different mechanisms of toxicity that are less responsive to probiotic intervention. A study conducted by Fei et al. (2019) identified an association between gut microbiota composition and chemotherapy-induced diarrhea in patients colorectal cancer receiving capecitabine and oxaliplatin based regimen. The results showed that patients who presented diarrhea had lower microbial diversity and richness compared to the control group, along with a predominance of *Klebsiella pneumoniae* what reinforce the influence of gut microbiota on gastrointestinal toxicity suggesting that microbiome modulation could represent a promising approach to mitigating this adverse effect of chemotherapy.

The heterogeneity of our patient population, including differences in baseline gut microbiota composition, chemotherapy regimens, and dietary factors, may have influenced treatment outcomes. Recent evidence suggests that personalized probiotic interventions based on individual microbiome profiles may be necessary to achieve consistent therapeutic benefits^20^ ^,^ ^21^.

One strength of this trial lies in its design as a double-blind, placebo-controlled study. This approach minimizes biases and enhances the reliability of the findings. The study also has some limitations. Our study had a relatively small sample size (28 patients), which may have limited the statistical power to detect significant differences. However, patients were followed for 90 days, with an average of six chemotherapy cycles, recruitment challenges are not uncommon in trials of this nature, and similar studies evaluating probiotic interventions have also faced difficulties in enrolling large cohorts of patients. Additionally, there was a higher proportion of patients with Stage III disease in the probiotic group (42.86%) compared to the placebo group (21.43%), which could have affected the overall response to the intervention. Additionally, irinotecan-based regimens were less commonly prescribed, though slightly more frequent in the probiotic arm (14.28%) than in the placebo arm (7.14%), what could influence the incidence of diarrhea, dietary habits were not recorded, which is an important limitation, as food significantly influences gut microbiota composition and function. Also, the microbiota profiling should be taken in consideration because without microbiome sequencing, it is difficult to confirm that probiotics successfully colonized the gut or modulated bacterial composition in order to be beneficial for CID prevention. The use of patient-reported bowel movement diaries introduces a potential for recall bias and variability in symptom reporting. Future studies should consider to increase sample sizes, diverse patient populations and analyze microbiome profiling that could help understand baseline gut microbiota and how it changes in response to probiotics could guide personalized interventions. Combining probiotics with prebiotics or using different strains such as *Lactobacillus rhamnosus* GG (LGG®) may enhance efficacy. Other approaches exploring multimodal interventions that integrate dietary modifications, probiotics, and pharmacologic agents may provide more effective CID management strategies.

## CONCLUSION

Our study did not find any significant benefit of probiotic supplementation tested in this study in reducing the incidence of CID in patients undergoing chemotherapy for gastrointestinal cancers. Further research is needed to identify better probiotic formulations, patient populations, and combination strategies to improve gastrointestinal toxicities in cancer patients undergoing chemotherapy.

## Data Availability

The research data are available in a public repository. Repository reference: de Souza, Gabriel (2025), “Efficacy of Probiotics in Preventing Chemotherapy-Induced Diarrhea in Gastrointestinal Cancer Patients”, Mendeley Data, V1, doi: 10.17632/bgbnkcgy9k.1. Link: https://data.mendeley.com/datasets/bgbnkcgy9k/1
